# Physiological Changes in the Levels of Anti-Cytokine Autoantibodies in Early Pregnancy Are Missing in Pregnant Women with Hashimoto's Thyroiditis

**DOI:** 10.1155/2023/5221658

**Published:** 2023-08-25

**Authors:** Szabina Erdő-Bonyár, Diána Simon, Anna Bajnok, Jasper Nörenberg, Tímea Serény-Litvai, Ákos Várnagy, Kálmán Kovács, Eszter Hantosi, Emese Mezősi, Tímea Berki

**Affiliations:** ^1^Department of Immunology and Biotechnology, Clinical Center, University of Pécs Medical School, Pécs, Hungary; ^2^National Laboratory on Human Reproduction, University of Pécs, Pécs, Hungary; ^3^Szentágothai Research Centre, University of Pécs, Pécs, Hungary; ^4^Department of Obstetrics and Gynecology, Clinical Center, University of Pécs Medical School, Pécs, Hungary; ^5^First Department of Internal Medicine, Clinical Center, University of Pécs Medical School, Pécs, Hungary

## Abstract

T helper type 1 (Th1) and inflammatory cytokines play essential roles in early pregnancy and also in the pathogenesis of Hashimoto's thyroiditis (HT). Changes in the serum level of autoantibodies to cytokines, which may be able to modulate their availability and actions have been described in several autoimmune disorders. Yet, no data are available on anti-cytokine autoantibodies either during early pregnancy or in patients with HT. The aim of the study was to measure autoantibodies to inflammatory-, Th1- and Th22-cytokines in serum samples in healthy pregnancy (HP) and in pregnant women with HT (HTP). As pathological autoantibodies are hallmarks of HT, in addition we also measured anti-B-cell activator factor (BAFF) autoantibodies. The measurement was carried out with a Luminex multiplex assay and the Luminex MAGPIX Instrument, age-matched healthy women (HC) and women with HT (HT) were used as controls. In the first trimester of HP, anti-TNF*α*, anti-IL-8, and anti-IFN*γ* autoantibodies were significantly decreased, while autoantibodies to BAFF were significantly elevated compared to the HC. However, these alterations were not present in the HTP. Moreover, the levels of autoantibodies to IL-22 and TNF*α* were significantly increased in HTP compared to the HP. All differences in the levels of the investigated autoantibodies could be detected in the first trimester of pregnancies except for anti-IL-22 autoantibodies. According to our results we can conclude that alterations in the levels of autoantibodies to inflammatory and Th1 cytokines are physiological in the first trimester of pregnancy and their disturbance can be associated with autoimmune conditions such as HT.

## 1. Introduction

Autoantibodies to cytokines are proposed to be able to regulate the effects of cytokines both in healthy individuals (HC) and in various autoimmune diseases [1, 2]. There are many divisions of cytokines and tumor necrosis factor-*α* (TNF*α*), interleukin-6 (IL-6), and interleukin-1 (IL-1) belong to pro-inflammatory cytokines [3] and these cytokines are disturbed in various patients, in COVID-19 as well as in cancer and they represent negative prognostic markers or the inflammation [4, 5]. Inflammatory and T helper type 1 (Th1) cytokines, for example TNF*α* and interferon-*γ* (IFN*γ*) are considered to be the central players during early pregnancy. This Th1 dominance seems to benefit the invasion of trophoblasts [6] and interleukin-22 (IL-22) promotes their proliferation at the maternal–fetal junction [7]. Hashimoto's thyroiditis (HT) have been describe to be a Th1-related autoimmune disorder; however, according to latest studies, interleukin-17 (IL-17) and IL-22-producing Th17 cells might have a vital role in the development of HT [8]. Moreover, another distinct subset of Th cells has been identified and named Th22 cells after their main effector cytokine IL-22 [9]. Only scarce data are available on the role of IL-22, a key cytokine produced by both Th17 and Th22 cells, in the development of HT. However, the elevated level of IL-22 was measured in the patients with HT compared to the HC in most studies [10]. HT has been described as a T-cell related disorder [11]. Nevertheless, the presence of diseases specific autoantibodies against thyroid antigens leading to thyroiditis implies the significance of B cells in the development of HT [12]. B-cell activator factor (BAFF) is vital for B-cell homeostasis, and higher levels of BAFF might support the pathogenesis of autoimmune diseases by disrupting the B-cell tolerance [13]. Elevated levels of BAFF were measured in serum samples of patients with HT compared to the HC [14, 15]. In contrast, serum BAFF levels steadily decline during pregnancy [16]. Anti-cytokine autoantibodies have been described to modify the effects of cytokines in several ways. However, no studies have investigated the anti-cytokine autoantibodies either during pregnancy or in patients with HT. Consequently, we aimed to measure serum levels of autoantibodies to Th1 and Th22-associated cytokines, inflammatory cytokines, and BAFF to evaluate the physiological and pathological changes in the anti-cytokine autoantibody network during pregnancy.

## 2. Material and Methods

### 2.1. Enrolled Individuals

Thirteen healthy pregnant women (HP), 9 pregnant women with HT (HTP), 13 women with HT (HT), and 10 healthy age-matched women (HC) were enrolled in this nonrandomized study. All selected subjects were women aged between 20 and 40 years. The HT and HTP subjects were antithyroid antibody positive and euthyroid patients. None of the participants had a concomitant disease or current infection. Additional inclusion criteria for pregnant women were normal pregnancy and attending regular prenatal care. Exclusion criteria for the pregnant women were significant obesity (BMI > 35 kg/m^2^), higher TSH (>4.2 mU/L), twin pregnancy, gestational diabetes, preeclampsia, toxemia, smoking, fetal abnormalities detected on ultrasound screening and preterm birth. Peripheral blood samples were taken in the first trimester (Week 13–14) and third trimester (Week 33–34) of pregnancy in the pregnant groups (HP and HTP). Following the approval of the Regional Research Ethics Committee of the Medical Center, University of Pécs (RIKEB 5913/2015), all participants have agreed in writing to take part in the study.

### 2.2. Anti-Cytokine Autoantibody Measurements

The MILLIPLEX Map Human Cytokine Autoantibody Panel (HCYTAAB-17K, Merck KGaA, Darmstadt, Germany) was used to measure the serum levels of anti-cytokine autoantibodies (mean fluorescence intensity, MFI) according to the manufacturer's protocol. In brief, 25 *μ*L of each 1 : 100 diluted serum samples, standards and controls with equal volumes of assay buffer and fluorescent-coded magnetic bead mixture coated with a specific antigen for anti-cytokine antibodies were added to the appropriate well of a 96-well plate provided with the kit and incubated overnight at 2–8°C for autoantibodies to bind to the relevant beads. After three rounds of washing, 50 *μ*L of the reported molecule, phycoerythrin-conjugated anti-IgG antibody was added and incubated for 90 min to complete the reaction on the surface of each bead, followed by the three washing steps. Each bead was identified and the result of the bioassay of each bead was measured based on the fluorescent reporter signals with the Luminex MAGPIX instrument (Luminex Corporation, Austin, TX, USA). Data were analyzed using the Belysa Immunoassay Curve Fitting Software (Merck KGaA, Darmstadt, Germany) per the manufacturers' instructions.

### 2.3. Statistical Analysis

For statistical assessment, the SPSS v. 27.0 statistical software package (IBM, Armonk, NY, USA) was used with Kruskal–Wallis and Mann–Whitney *U*-test, where *p* values < 0.05 were regarded as significant.

## 3. Results

### 3.1. The Decrease in Anti-IFN*γ* Autoantibody Level in HP Is Not Present in HTP

**Figure 1:**
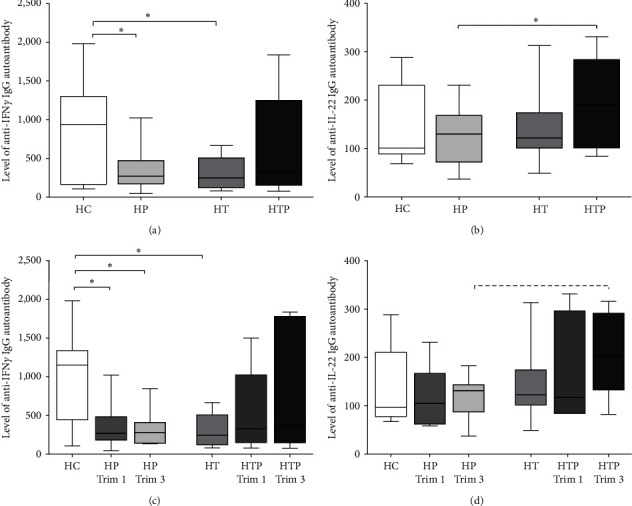
Investigation of autoantibodies against two Th1-related cytokines in HC, HP, HT, and HTP. The serum levels of anti-IFN*γ* and anti-IL-22 autoantibodies were tested in all samples of HC (*n* = 10), HP (*n* = 23), HT (*n* = 13), HTP (*n* = 14) (a, b) and examined in the first and third trimester samples separately in HP (*n*_trim1_ = 13, *n*_trim3_ = 10) and HTP (*n*_trim1_ = 8, *n*_trim3_ = 6) (c, d). The boxes represent the interquartile ranges (IQR), the horizontal lines the medians and the whiskers the lowest and highest values.  ^*∗*^*p* < 0.05. The dashed lines show the trends, where *p* is between 0.05 and 1.

First, we analyzed the autoantibodies against two Th1- and Th22-related cytokines (IFN*γ* and IL-22) in the sera of the four investigated groups. The autoantibody levels against IFN*γ* were significantly lower in HP than in HC (*p*=0.049), but this decline was not observed in HTP. No difference in anti-IFN*γ* autoantibody levels was found between HT and HTP, but HT had significantly reduced levels compared to the HC (*p*=0.035) (Figure 1(a)). A significant elevation in anti-IL-22 antibody level was detected in HTP compared to the HP (*p*=0.046); however, no significant differences were found between the other groups (Figure 1(b)). When first trimester and third trimester samples were examined separately, anti-IFN*γ* autoantibody levels in HP were significantly diminished in both the first and third trimester compared to the HC (*p*=0.027 and 0.028), while in HTP, this decrease was not detected (Figure 1(c)). A tendentious increase in the IL-22 autoantibody level was seen in the third trimester in HTP compared to the HP (Figure 1(d)).

### 3.2. Autoantibodies to Inflammatory Cytokines Are Diminished in HP but Not in HTP

**Figure 2:**
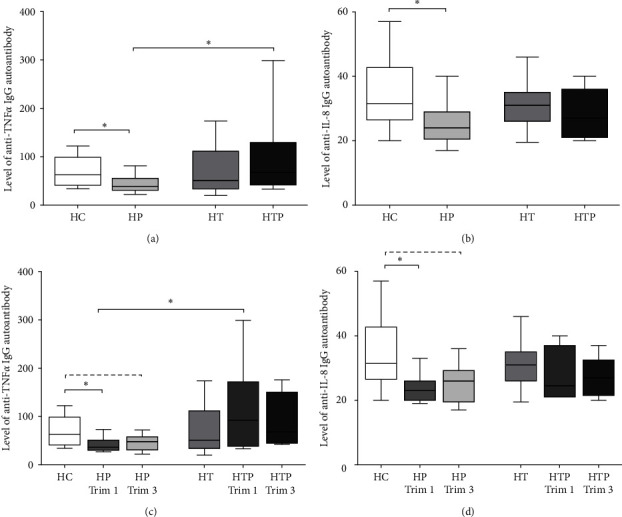
The difference in the serum level of autoantibodies against two pro-inflammatory cytokines. The levels of anti-TNF*α* and anti-IL-8 autoantibodies were measured in all samples of HC (*n* = 10), HP (*n* = 23), HT (*n* = 13), HTP (*n* = 14) (a, b) and in the first and third trimester samples separately in HP (*n*_trim1_ = 13, *n*_trim3_ = 10) and HTP (*n*_trim1_ = 8, *n*_trim3_ = 6) (c, d). The boxes represent the interquartile ranges (IQR), the horizontal lines the medians and the whiskers the lowest and highest values.  ^*∗*^*p* < 0.05. The dashed lines show the trends, where *p* is between 0.05 and 1.

Next, we investigated the autoantibodies against two pro-inflammatory cytokines (TNF*α* and IL-8) in HP and HTP compared to HC and HT. We found lower levels of anti-TNF*α* and anti-IL-8 autoantibodies in HP than in HC (*p*=0.023 and 0.021), but these decreases were not detectable in HTP compared to the HC. In addition, the amount of anti-TNF*α* autoantibodies showed a significant raise in HTP compared to the HP (*p*=0.009). No differences in the levels of anti-TNF*α* and anti-IL-8 autoantibodies were found between the other investigated groups (Figures 2(a) and 2(b)).

When we examined the autoantibodies against inflammatory cytokines in the first and third trimester samples of HP and HTP compared to the HC, we found that the previously observed differences in anti-TNF*α* and anti-IL-8 autoantibody levels were due to alterations detected mainly in the first trimester. In HP, their levels decreased significantly in the first trimester (*p*=0.022 and 0.018) and tended to diminish in the third trimester compared to the HC, but these reductions were not detectable in the HTP. Moreover, the anti-TNF*α* level was higher in HTP than in HP in the first trimester (*p*=0.044) (Figures 2(c) and 2(d)).

### 3.3. The Increase in the Level of Anti-BAFF Autoantibodies in HP Is Missing in HTP

**Figure 3:**
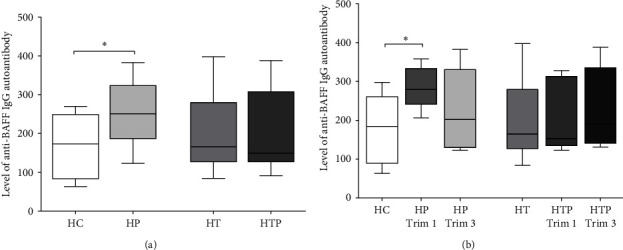
Measurements of autoantibodies against BAFF in HC, HP, and HTP serum samples. Levels of anti-BAFF autoantibodies were investigated in all samples of HC (*n* = 10), HP (*n* = 23), HT (*n* = 13), HTP (*n* = 14) (a) and separately in the first and third trimester samples in HP (*n*_trim1_ = 13, *n*_trim3_ = 10) and HTP (*n*_trim1_ = 8, *n*_trim3_ = 6) (b). The boxes represent the interquartile ranges (IQR), the horizontal lines the medians and the whiskers the lowest and highest values.  ^*∗*^*p* < 0.05.

We also examined the levels of autoantibodies against BAFF and showed that anti-BAFF autoantibodies were present in higher levels in HP than in HC (*p*=0.043), but this difference was not present in HTP. We did not see any difference in its level between the other tested groups (Figure 3(a)). Our results were similar in the trimester-separated groups; namely, the level of anti-BAFF autoantibody in the first trimester HP samples was significantly increased compared to the HC (*p*=0.021), but this elevation was absent in HTP (Figure 3(b)).

## 4. Discussion

The dominance of the Th1-type immune response has been described in early pregnancy. IFN*γ* is a Th1-associated cytokine crucial for establishing and maintaining early pregnancy, mediating endometrial vascular remodeling, and angiogenesis [17]. Yet, studies have reported different directional changes in the IFN*γ* serum levels through pregnancy [18–20]. HT is described as a Th1-mediated autoimmune disease, and significant production of IFN*γ* by lymphocytes infiltrating thyroid tissues has been reported, which locally contributes to the destruction of thyroid tissues [21]. However, data on the serum level of IFN*γ* are inconsistent in the literature [22, 23]. The decreased anti-IFN*γ* autoantibody level in HP might be a part of regulatory mechanism during a healthy pregnancy, which could be impaired in HTP. IL-22 is a cytokine with ability to upregulate innate immune responses, promote tissue regeneration, and help to maintain the tissue integrity. The utilities of IL-22 in maternal–fetal immunity during pregnancy are poorly understood. In pregnant women, helper T cells of the decidua produced a higher amount of IL-22 than the helper T cells of peripheral blood. In pregnancies resulting in childbirth, IL-22 and IL-4 secreting helper T cells were found to be predominant in the decidua [24]. The receptors of IL-22 (IL-22R) are expressed in the placenta, and the IL-22/IL-22R pathway could have a basic role in supporting the survival of the trophoblasts and sustaining pregnancy [25]. Additionally, T-cell mediated inflammatory disorders have been associated with the activation of the IL-22/IL-22R system [26]. In untreated HT patients who were newly diagnosed increased level of serum IL-22 accompanied by a higher expression of IL-22 in the thyroid glands were reported compared to HC [27]. The presence of antibodies against IL-22 has only been reported in the psoriasis [28] and in the patients with autoimmune polyglandular syndromes [29]. Our results show an elevated level of anti-IL-22 autoantibody in HTP compared to the HP, but it was not higher in HT compared to the HP. Thus, the increase in anti-IL-22 antibody levels appears to be related to the pregnancy of women with HT.

TNF*α* is a critical mediator of inflammatory processes, and its involvement has been described in regulating vital biological functions, including cell proliferation, production of other cytokines, and apoptosis [4, 5, 30]. TNF*α* also plays a crucial role in the inflammatory mechanism that regulates implantation, placentation, trophoblast cell survival, and pregnancy outcome [31, 32]. However, it has also been suggested that elevated levels of inflammatory cytokines, especially TNF*α*, may contribute to several obstetric abnormalities [33]. The literature on the changes in TNF*α* levels during pregnancy is not consistent; its increase, decrease, and stability have been described [18, 20, 32], while the serum level of TNF*α* was found to be elevated in patients with HT [22, 34, 35], and lymphocytes infiltrating thyroid tissues were shown to produce TNF*α* [21]. Sjöwall et al. [36] reported that the serum level of anti-TNF*α* autoantibodies in patients with systemic lupus erythematosus inversely correlated with the disease severity, suggests the possible role of anti-TNF*α* autoantibodies in regulating the availability and bioactivity of TNF*α*. Therefore, our finding of a lower level of anti-TNF*α* autoantibodies in first trimester HP might be the part of a regulatory mechanism resulting in a higher activity of TNF*α* necessary for the inflammatory processes in this period of pregnancy. Interestingly, the level of anti-TNF*α* autoantibody is elevated in HTP compared to the HP indicating a possible increase in the activity of TNF*α* in HTP. IL-8 is also an inflammatory cytokine, which is responsible for the recruitment and activation of immune cells, especially neutrophils, to sites of inflammation [37]. IL-8 plays a role in mediating angiogenesis by stimulating endothelial cell proliferation and survival [38], and angiogenesis is an essential process during the fetal development [39]. Studies have found elevated levels of IL-8 in the first trimester compared to the second, reflecting decreased inflammatory processes in the second trimester [18]. IL-8 mRNA expression has been described in thyroid tissue samples of HT patients, thus IL-8 may contribute by chemotactic stimulation to drive the extravasation and migration of lymphocytes into the thyroid gland [40]. However, IL-8 serum level was not elevated in the HT patients [41]. The presence of autoantibodies against IL-8 has also been described in the serum of healthy individuals [42], but their higher prevalence in the serum of patients with rheumatoid arthritis has been found, which correlated with the disease severity [43]. The presence of anti-IL-8 autoantibodies in the alveolar fluid of patients with adult respiratory distress syndrome has also been associated with the increased mortality [44]. These findings propose that anti-IL-8 autoantibodies have a regulatory role in the inflammatory processes. However, we measured a lower level of anti-IL-8 autoantibodies in HP than in HC, indicating that functional, free IL-8 may be a key player of inflammation during the first trimester in the physiological pregnancy. Nonetheless, we did not detect a decline in the level of anti-IL-8 autoantibodies in HTP, suggesting a disturbance in the regulation of these autoantibodies in HTP.

It has recently been reported that the placenta secretes BAFF. Both in early and term pregnancies BAFF accompanied by its receptor could be detected in the placenta [45]. Trophoblasts and stromal cells of the decidua were shown to express BAFF. Furthermore, BAFF-R secretion was suggested to be an inherent property of decidual stromal cells [45], and this soluble BAFF-R might inhibit the functions of macrophages. Therefore, the BAFF system might play a vital role in the successful pregnancies. BAFF might be helpful in the prediction of disease severity in HT [46]. Interestingly, in the B cells of patients with HT, BAFF-R were not expressed at higher levels compared to the HC. However, BAFF and BAFF-R were shown to be expressed in the thyrocytes derived from patients with HT, suggesting the possible involvement of BAFF and its receptors in the pathogenesis of HT [47]. Anti-BAFF autoantibodies regulate the accessibility and effects of BAFF [1]. Autoantibodies to BAFF have been detected in the serum samples of HC, and these autoantibodies were increased in the patients with systemic autoimmune diseases [48, 49]. Thus, the higher levels of autoantibodies to BAFF we measured in HP could be a response to elevated BAFF, which regulatory mechanism might be impaired in the HTP.

## 5. Conclusion

According to our knowledge, we were the first to measure anti-cytokine autoantibodies during pregnancy and in patients with HT. Our study has the limitation that the number of enrolled individuals was small, which limits the determination of the clinical significance of the obtained alterations in the levels of the autoantibodies to cytokines. However, we can conclude that we found differences in the levels of autoantibodies to IFN*γ*, TNF*α*, and IL-8 between HP and HC in the first trimester of pregnancy, which is consistent with the known pivotal role of inflammatory and Th1 cytokines in the early pregnancy. Additionally, the elevated anti-TNF*α* and anti-IL-22 autoantibody levels in HTP compared to HP may indicate immunological alterations associated with the pregnancy of women with HT.

## Data Availability

The data that support the findings of this study are available from the authors (Szabina Erdő-Bonyár and Diána Simon) upon reasonable request.
